# Variation in signal–preference genetic correlations in *Enchenopa* treehoppers (Hemiptera: Membracidae)

**DOI:** 10.1002/ece3.1567

**Published:** 2015-06-19

**Authors:** Kasey D Fowler-Finn, Joseph T Kilmer, Allysa C Hallett, Rafael L Rodríguez

**Affiliations:** 1 Behavioral and Molecular Ecology Group, Department of Biological Sciences, University of Wisconsin–MilwaukeeMilwaukee, Wisconsin; 2 Department of Biology, Saint Louis UniversitySaint Louis, Missouri

**Keywords:** Assortative mating, Fisher’s runaway, male–female genetic covariance, preference function, vibrational communication

## Abstract

Fisherian selection is a within-population process that promotes signal–preference coevolution and speciation due to signal–preference genetic correlations. The importance of the contribution of Fisherian selection to speciation depends in part on the answer to two outstanding questions: What explains differences in the strength of signal–preference genetic correlations? And, how does the magnitude of within-species signal–preference covariation compare to species differences in signals and preferences? To address these questions, we tested for signal–preference genetic correlations in two members of the *Enchenopa binotata* complex, a clade of plant-feeding insects wherein speciation involves the colonization of novel host plants and signal–preference divergence. We used a full-sibling, split-family rearing experiment to estimate genetic correlations and to analyze the underlying patterns of variation in signals and preferences. Genetic correlations were weak or zero, but exploration of the underlying patterns of variation in signals and preferences revealed some full-sib families that varied by as much as 50% of the distance between similar species in the *E. binotata* complex. This result was stronger in the species that showed greater amounts of genetic variation in signals and preferences. We argue that some forms of weak signal–preference genetic correlation may have important evolutionary consequences.

## Introduction

The evolution of sexual traits such as advertisement signals and ornaments is characterized by three general patterns: Sexual traits represent some of the most spectacular and elaborate structures and behaviors in nature; they are frequently the most divergent aspects of the phenotype among recently diverged species; and they often show a high degree of correspondence with mate preferences across populations and species (Darwin 1871; West-Eberhard 1983, 2014; Eberhard 1985, 1996; Andersson 1994; Gerhardt and Huber 2002; Greenfield 2002; Coyne and Orr 2004; Mendelson and Shaw 2005; Arnegard et al. 2010; Prum 2010; Safran et al. 2012; Rodríguez et al. 2013a). Extensive theoretical and empirical work has established sexual selection due to mate choice as a main agent in the evolution of sexual traits (Kirkpatrick and Ravigné 2002; Andersson and Simmons 2006; Kokko et al. 2006; Rodríguez et al. 2013a). However, the connection between the within-population dynamics that are involved in mate choice with the among-population patterns of diversification and speciation is less clearly understood (Kokko et al. 2006; Shaw and Lesnick 2009).

The simplest within-population mechanism that can generate coevolution between signals and preferences is a genetic correlation between them. In principle, for a signal–preference genetic correlation to arise, all that is required is the presence of genetic variation in the signal, genetic variation in the preference, and an initial cause of assortative mating (Fisher 1930). These three simple conditions are common in nature. Genetic variation in signals and preferences is widespread (Bakker and Pomiankowski 1995; Chenoweth and McGuigan 2010; Prokuda and Roff 2014), and assortative mating may arise for various reasons – including selection on females to obtain direct and/or indirect benefits from their mates, and the co-option of sensory biases (Fisher 1930; West-Eberhard 1983, 2014; Mead and Arnold 2004; Kokko et al. 2006; Rodríguez 2009). Once a genetic correlation between a signal and preference has been established, selection on the signal also exerts indirect selection on the preference, and evolutionary change in the preference further exerts selection on the signal. Thus, signal–preference genetic correlations result in a self-reinforcing coevolutionary process (Fisherian selection) that promotes signal–preference elaboration and diversification (Fisher 1930; Lande 1981; Kirkpatrick 1982; Higashi et al. 1999; Mead and Arnold 2004). Because the above-mentioned starting conditions for Fisherian selection are typical of natural populations, it has been offered as the default mechanism of sexual selection and speciation (Fisher 1930; Prum 2010, 2012).

Despite its potential pervasiveness, Fisherian selection is controversial. This is in part because indirect selection on mate choice is expected to be weak and easily countered by costs of expressing mate choice (Kirkpatrick and Barton 1997; Servedio and Bürger 2014). However, few studies have compared the strength of selection favoring and opposing mate choice (Kokko et al. 2003; Prum 2012); some studies even suggest that indirect selection favoring mate choice may be stronger than direct selection opposing it (Head et al. 2005). Another reason for doubt about the biological importance of Fisherian selection is the difficultly of testing it empirically, and the common finding of weak or absent signal–preference genetic correlations in existing tests (Kirkpatrick and Ryan 1991; Andersson 1994; Bakker and Pomiankowski 1995; Greenfield et al. 2014) (but see Prum 2010). However, a recent review found that signal–preference genetic correlations are surprisingly common, being detected in over 60% of the studies that have tested for them (Fowler-Finn and Rodríguez 2015). A key variable explaining variation across studies in the detection of signal–preference correlations is the amount of genetic variation in the mate preference; in studies where genetic variation in the preference is medium–high, genetic correlations are detected ∼90% of the time, whereas this percentage is zero in studies where genetic variation in the preference is low–absent (Fowler-Finn and Rodríguez 2015). Genetic variation in the preference is, of course, one of the required conditions for signal–preference genetic correlations to be established (Fisher 1930; and see Lande 1981; Roff and Fairbairn 2014 on the role of the relative magnitude of the genetic variances in signals and preferences). Additionally, tests with different populations of the same species often vary in whether signal–preference correlations are detected (Bakker and Pomiankowski 1995; Greenfield et al. 2014; Fowler-Finn and Rodríguez 2015). This variation may be due to differences among populations and/or experimental conditions influencing the expression of genetic variation (Bakker and Pomiankowski 1995; Fowler-Finn and Rodríguez 2015). Thus, studies of Fisherian selection should not only test for signal–preference genetic correlations, but also seek to explain variation in their presence and strength.

Here, we explore variation in signal–preference genetic correlations in the *Enchenopa binotata* species complex of treehoppers (Hemiptera: Membracidae). We also compare the range of within-population genotypic variation in signals and preferences to among-species differences in the species complex. As in many herbivorous insects, speciation in the *E. binotata* complex is associated with shifts to novel host plants and with divergence in the treehoppers’ plant-borne vibrational communication systems (Rodríguez et al. 2006; Cocroft et al. 2008). Signals and preferences have coevolved in the complex, with signal–preference correspondence being a function of the strength of mate preferences (Rodríguez et al. 2006, 2013a). Thus, the *E. binotata* complex provides an exceptional opportunity to study how population-level processes result in speciation. In this study, we focus on the dominant frequency of male signals and on the mate preference for it – signal frequency is the adult phenotype that most diverges across the *E. binotata* complex, and the signal trait for which females show the strongest preferences (Rodríguez et al. 2006, 2013a; Cocroft et al. 2010; Sullivan-Beckers and Cocroft 2010).

We worked with two members of the *E. binotata* complex, selected opportunistically but with the aim to explore variation in signal–preference genetic correlations and in the underlying patterns of genetic variation in signals and preferences. A robust test of such relationships would require broad comparative sampling, but we consider that our study offers a useful qualitative comparison between the two study species.

**Figure 1:**
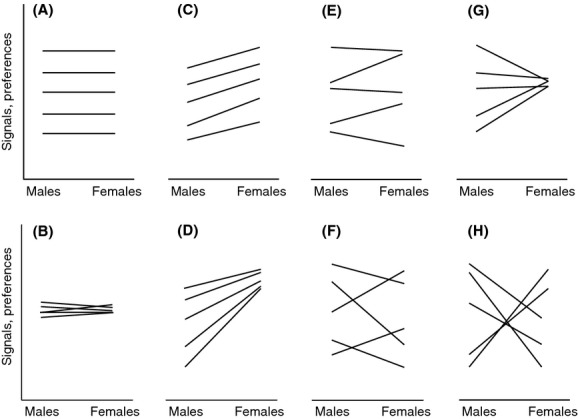
Illustration of potential forms of genetic variation in the signal–preference relationship. In each panel, each line indicates signal and peak preference values for a genotype. (A) Strong signal–preference genetic correlation: among-genotype differences in the *y*-axis intercept, with all genotypes showing strong signal–preference correspondence (parallel lines), so that *r *=* *1. (B) *r* is zero or very weak due to lack of overall genetic variation in signals and preferences. (C) *r *=* *1 or nearly so. (D) 0 < *r *<* *1 due to lower amounts of genetic variation in the preference, which also results in genotype-level signal–preference mismatch. (E) 0 < *r *<* *1 due to genotype-level signal–preference mismatch. (F) *r *≪ 1 due to genotype-level signal–preference mismatch, but there are still some genotypes (those with phenotypes at the extremes of the range) that remain distinct from others in their signal–preference relationship. (G) *r* ≪ 1 due to lack of genetic variation in the preference, which also results in genotype-level signal–preference. (H) *r* ≪ 0 due to strong genotype-level signal–preference mismatch. NB: Negative signal–preference genetic correlations have been documented (Bakker and Pomiankowski 1995; Greenfield et al. 2014). In cases of population-level correspondence between mean signal and preference values, stabilizing sexual selection due to mate choice is predicted (A, B, E–H). By contrast, in cases of population-level signal–preference mismatch, directional sexual selection is predicted (C, D).

We conducted quantitative genetics rearing experiments with the two species and two complementary methods of analysis. Both methods involve mixed models, but offer different advantages. We used the animal model (Wilson et al. 2010) to obtain high-quality estimates of the amount of genetic variation in signals and preferences and of signal–preference genetic correlations. We then analyzed the patterns of expression of genetic variation in signals and preferences that underlie the signal–preference relationship using a second method. This second method is modified from Gray and Cade (2000) according to Fry (1992), and it views the relationship between a genotype’s signal and preference values as a reaction norm (Fig.1) (see also Roff 1997). This “reaction norm approach” allows us to apply the framework for analysis of variation in reaction norms (i.e., genotype × environment interaction, or G × E) (Fry 1992; Hunt et al. 2004) to the various forms that the signal–preference relationship may take (Fig.1). It also allows testing for population-level correspondence between signals and preferences, which provides information about the form of sexual selection in the population (Fig.1).

**Figure 2:**
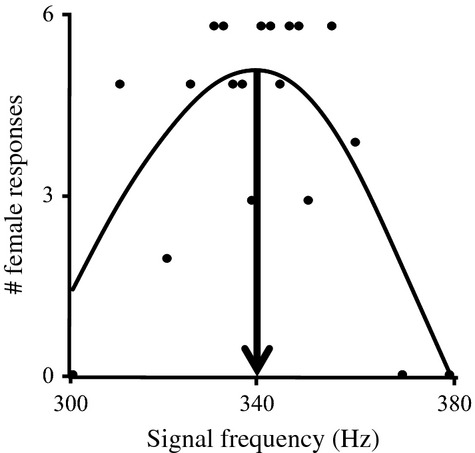
Example of a female mate preference function for male signal frequency for one female individual from *Enchenopa binotata “*Ptelea.” The peak preference (arrow) is derived from the cubic spline (curved line) that fits the raw data (data points), and corresponds to the signal frequency to which this female had the strongest response.

For both analyses, the amount of genetic variation in signals and preferences is a fundamental predictor of the likelihood of signal–preference correlations being established (Fisher 1930; Bakker and Pomiankowski 1995; Fowler-Finn and Rodríguez 2015). Because genetic variation in mate preferences can be particularly challenging to measure (Chenoweth and Blows 2006; Rodríguez et al. 2013b), we placed emphasis on obtaining high-quality descriptions of individual mate preferences. Mate preferences are function-valued traits (Meyer and Kirkpatrick 2005; Stinchcombe and Kirkpatrick 2012), meaning that they are expressed as variation in sexual response along variation in signals (Wagner et al. 1995; Ritchie 1996; Wagner 1998). We view individual female preference function as the traits of interest (Fowler-Finn and Rodríguez 2013; Rodríguez et al. 2013b), and we extract from each function the key measure for testing for signal–preference correlations: the peak of the preference, which is the signal trait value eliciting the highest response (Fig.2).

Finally, we examine the range of among-genotype variation in signals and preferences against the background of species differences in the *E. binotata* complex.

## Materials and Methods

### Study species and experimental rearing design

Our two study species were the members of the *E. binotata* complex that live on *Ptelea trifoliata* (Rutaceae) host plants in Missouri, USA, and on *Viburnum lentago* (Caprifoliaceae) host plants in Wisconsin, USA. Most species in the complex have not been described (Hamilton and Cocroft 2009), and so we refer to our study species by the names of their host plants: *E. binotata “*Ptelea” and *E. binotata “*Viburnum.” At our Wisconsin study site (Saukville), there are two *E. binotata* species that live on *V. lentago* plants. Species in the complex are easily distinguished by the frequency of the male signals (Wood and Guttman 1982; Lin and Wood 2002; Rodríguez et al. 2004; Cocroft et al. 2008; Cocroft et al. 2010; McNett and Cocroft 2008; Hamilton and Cocroft 2009). We used the species that lives on *V. lentago* that has a mean male signal frequency of ∼165 Hz (vs. ∼315 Hz in the other species). We kept voucher specimens in 95% EtOH in the Rodríguez Laboratory collection.

We used a full-sib, split-family rearing design (Roff 1997) to partition variation in signals and preferences among components for family (as a proxy for genotype), rearing environment within family, and sex. With this design, our estimates include additive and nonadditive components of variation and do not predict the short-term response to selection (Roff 1997; Lynch and Walsh 1998). However, with the growing realization that both additive and nonadditive genetic components of variation are important for evolution (Day and Bonduriansky 2011) and that genetic and environmental inputs during development can expose genetic variation to selection (West-Eberhard 2003, 2005; Suzuki and Nijhout 2006; Gerhart and Kirschner 2007; Barrett and Schluter 2008), our main interest was in variation among and within families.

To establish full-sib families, we collected mated females in late summer of 2010 and 2011 at the UWM Field Station (Saukville, WI) for *E. binotata “*Viburnum,” and in the late summer of 2011 and 2012 in Columbia, MO for *E. binotata “*Ptelea.” Note that females in the *E. binotata* complex mate only once (Wood 1993; Sullivan-Beckers and Cocroft 2010). Consequently, a female’s brood constitutes a full-sib family. We allowed the mated females to oviposit on potted host plants, one female per plant. Upon nymph eclosion the following spring, we divided each brood into half and placed each half on a different rearing plant. We reared the treehoppers on potted plants of standard size (∼0.5–0.9 m tall), condition, and phenology. We selected broods large enough to allow for ∼20 nymphs on each rearing plant (40 nymphs/family). This resulted in ∼25 families for each species at the start of the experiment. We reared the treehoppers in the UWM greenhouse at temperatures that corresponded to outside temperatures during the late spring/early summer. On very warm days, we used shades and vents to prevent extreme heat, and on very cloudy days, we used supplemental lighting. Upon the final molt to adulthood, we separated males and females and placed them on fresh rearing plants (two replicates per sex per family). This allowed us to control the experience of adults with the opposite sex (Fowler-Finn and Rodríguez 2012a,b; Rodríguez et al. 2013c) and ensured that females had not mated and were sexually receptive at the time of testing.

We recorded the males at the peak of their signaling activity, 2–6 weeks post-adult molt. We assayed the females at the peak of their receptivity, 6–8 weeks post-adult molt.

For all analyses, we used only families with a minimum of two individuals per sex per replicate. Measuring heritability requires only measurements from one sex per family, whereas estimating signal–preference correlations requires measurements for both sexes for each family. Thus, our final sample sizes for heritability were larger than those for the correlations (*E. binotata* “Ptelea”: median sample of *N* = 10 females/family and *N* = 13 males/family, *N* = 26 families for heritability, *N* = 15 families for the correlation; *E. binotata “*Viburnum”: median sample of *N* = 10 females/family and *N* = 14 males/family, *N* = 31 families for heritability, *N* = 13 families for the correlation).

### Description of male signals

*Enchenopa* males fly from plant to plant, signaling as they search for females (Cocroft et al. 2008). Thus, they often commence to signal when placed on a stem of their host plant. We used this behavior to induce males to signal by placing them, one by one, on the stem of a potted recording plant. If a male did not signal within 2 min, we played a “primer” stimulus consisting of a recording of a live male–female duet. This induces males to signal, but does not change signal frequency. If a male did not signal on a given test day, he was placed back onto his plant and retested a few days later.

We recorded signals using a laser vibrometer (Polytec CLV 2534; Polytec Inc., Auburn, MA). This no-contact method allows recording substrate-borne vibrational signals without altering the signal-transmission properties of the substrate. We isolated the recording setup from building vibrations using a large ∼135-kg iron plank placed on bicycle tire inner tubes on the experimental table surface. The table had rubber pads under its legs. We recorded the laser output and analyzed the recordings with the program AUDACITY (v. 1.2.4; http://audacity.sourceforge.net/) on an iMac computer.

### Description of female mate preferences

We used vibrational playback experiments to describe female mate preferences. To obtain an assay of female response, we took advantage of the duetting exchange that facilitates pair formation in *Enchenopa* (Rodríguez and Cocroft 2006). When males signal, females respond to the signals that they find attractive with their own duetting signals, and this provides a convenient and biologically relevant assay of mate preference. The number of responses a female gives to a given stimulus indicates the level of her preference (Rodríguez et al. 2004, 2012; Fowler-Finn and Rodríguez 2012b). For each female, in random order, we played back 19 stimuli spanning and slightly exceeding the species range of signal frequency, with the other signal parameters set to the species mean. Each stimulus consisted of a bout of signals corresponding to the typical structure for the species (4 signals/bout for *E. binotata* “Viburnum” and 6 signals/bout for *E. binotata “*Ptelea”). Stimuli varied by ± 2, 4, 6, 8, 10, 15, 20, 30, and 40 Hz in either direction from the species mean (338 Hz for *E. binotata “*Ptelea” and 185 Hz for *E. binotata “*Viburnum”).

We generated stimuli and controlled playbacks with custom scripts written in MATLAB v. 7.5.0 (The Mathworks, Inc., Natick MA) (scripts available upon request). The stimuli were imparted to the stem of a potted playback plant at an amplitude of 0.15 mm s^−1^ with a piezoelectric controller and actuator (Thorlabs, Newton, NJ). We recorded the playbacks and female responses with the laser vibrometry system described above.

For each female, we constructed a full preference function using cubic spline regressions. Cubic splines make no assumptions about the shape of the function other than smoothness (Schluter 1988). We generated splines in R v. 3.0.2. (R Development Core Team 2008) using the mgcv package, *gam* function, and a custom-written script (available upon request). We then optimized the smoothing parameter for each individual female. From each individual preference function, we measured the peak preference – the stimulus frequency that elicited the highest response (Fig.2; Fowler-Finn and Rodríguez 2012a,b, 2013; Rodríguez et al. 2013b).

### Testing for genetic variation in overall female mate preference functions

We constructed a linear mixed model in JMP 7.0.1 (SAS Institute, Cary, NC). The dependent variable was the number of female responses to the playback stimuli (see above). We included the following as independent variables: family; rearing plant replicate nested within family; individual female ID nested within replicate and family; linear and quadratic terms for stimulus frequency; and the interaction between the linear and quadratic terms with family. Family, replicate, individual ID, and their interactions with other terms were random effects. Female preferences in *E. binotata* are curvilinear, with peak preferences at intermediate signal frequencies (Fig.2; Rodríguez et al. 2006; Fowler-Finn and Rodríguez 2013; Rodríguez et al. 2013b). Thus, genetic variation in the preference functions is indicated by a significant family × quadratic stimulus interaction (Rodríguez et al. 2013b).

### Testing for genetic variation in female peak preferences and male signals

We implemented the animal model in R using the MCMCglmm package (Hadfield 2010), following Wilson et al. (2010). To adjust our full-sib split-family design to the framework of the animal model, we coded pedigrees with one sire and one dam per family and no relatedness among sires and dams. Our priors assumed that phenotypic variance was divided equally among the individual, replicate, and residual effects, with low degree of belief in the prior (Wilson et al. 2010). Varying the ratios of the priors did not substantially change the outcome of the model. Chain lengths were 1,000,000 iterations, with a burn-in of 500,000 iterations, and sampling every 500 iterations. All autocorrelation values were less than 0.001 by the end of the runs. We report heritability estimates with 95% confidence intervals (CIs) and estimate posterior distributions. NB: The bandwidths used to obtain the point estimates are 0.1× of the bandwidths used to generate the posterior distributions, as per the default in the MCMCglmm package (Hadfield 2010). We also report the coefficient of additive variance (CV_A_), calculated from the animal model variance estimates (CV_A_ = 100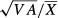 (Houle 1992)).

### Testing for signal–preference genetic correlations

We used two approaches to estimate signal–preference genetic correlations and explore the underlying patterns of variation in signals and preferences: the animal model approach and the reaction norm approach.

#### Animal model approach

We estimated genetic correlations between male signals and female peak preferences using the implementation of the animal model described above. To obtain these estimates, we set the residual covariance to zero, because any given individual has a value for either a signal or a preference, but not both (Roff and Wilson 2014). We report point estimates for the genetic correlations with their 95% CIs.

#### Reaction norm approach

We implemented this approach with a linear mixed model in JMP. We used a single dependent variable to represent female peak preference and male signal frequency, with an explanatory variable for sex (male/female) to indicate whether the data were for signal or preference (Gray and Cade 2000; Rebar and Rodríguez 2015). This codification permits analyzing the relationship between a genotype’s signal and preference values as a reaction norm (Fig.1; Roff 1997; Gray and Cade 2000). The model also included the following random explanatory variables: family, rearing plant replicate nested within family, and the family × sex interaction.

The reaction norm approach offers two ways to analyze genetic variation in the signal–preference relationship (Fry 1992). First, in the basic linear mixed model in JMP, the *F*-test for the family term is calculated as MS_family_ over a synthetic MS with components from replicate, the family × sex interaction, and the residual. The family × sex interaction tests for signal–preference mismatch among genotypes (nonparallel lines in Fig.1), which would indicate a signal–preference genetic correlation of *r *<* *1. The family term tested over the synthetic MS therefore tests for *r *>* *0. For example, in Figure1A,C, the family term would be significant and the family × sex interaction would be nonsignificant. In Figure1D,E, both terms would be significant because 0 < *r *<* *1 in spite of some mismatch among genotypes. But in Figure1F–H, only the interaction would be significant (cf. Roff 1997; Gray and Cade 2000; Rebar and Rodríguez 2015). Thus, the family term is a more sophisticated version of what Fry (1992) terms the “SAS model” (*F *= MS_family_/MS_interaction_). It also corresponds to the animal model estimates for signal–preference genetic correlations (Roff and Wilson 2014; see below).

Second, the family term can also be tested as *F *= MS_family_/MS_residual_ to ask about variation among families averaged across sexes – in a standard rearing experiment this would ask about genetic variation averaged across environments (Fry 1992; “Scheffé model”). Applied to our data, this test adds resolution to our exploration of variation in the signal–preference relationship. The family term tested over the residual MS would be significant whenever the SAS model returned significance, and it would also be significant in cases such as Figure1F–G. However, it would not be significant for Figure1H (cf. Fry 1992; Rodríguez et al. 2008). We were interested in recognizing cases where the signal–preference genetic correlation is weak or zero, but where there might nevertheless be assortative mating for subsets of genotypes (e.g., Fig.1F, top vs. bottom signal–preference lines). We consider that the ability to dissect the signal–preference relationship in this detail (i.e., distinguishing between cases A–H in Fig.1) makes the reaction norm approach a valuable complement to the animal model approach, even though the latter is more modern and avoids certain assumptions that the SAS model makes, such as equality of variances across sexes (or environments; Fry 1992; Roff 1997; Roff and Wilson 2014). Visualizing the data with reaction norms, as in Figure1, complements the analysis and makes any sex differences in genetic variance easy to identify and interpret. Further, our data partially meet the assumption of equal variances in the sexes (heritability was greater for signals than for preferences in both species, but because of greater within-family variation for preferences, rather than lower range of genotypic values; see below). Importantly, the key test of *F *= MS_family_/MS_residual_ does not make the assumption of equal variances (Fry 1992; Roff 1997).

We also use the main term for sex to test for population-level signal–preference correspondence or mismatch (e.g., Fig.1A vs. C). NB: In the JMP reaction norm model, the main term for sex is tested as MS_sex_ over a synthetic MS with components from the family × sex interaction, and the residual.

### Range of signal–preference genetic variation relative to species differences

We examined the range of within-population (among-family) variation in signals and preferences in relation to differences in signals and preferences between species in the *E. binotata* complex. To this end, we plotted family means for female peak preference and male signal frequency on a scatterplot showing the species means for the same traits. These “background” species values span the range of the lowest and highest known signal frequencies in the complex (Rodríguez et al. 2006; Cocroft et al. 2008, 2010).

## Results

### Testing for genetic variation in overall female mate preference functions

We found significant genetic variation in female mate preference functions in both species. The significant family × quadratic stimulus frequency term indicates family differences in the curvilinear shape of the preference functions (Table1).

**Table 1:** Test for genetic variation in female mate preference functions for male signal frequency in two species of the *Enchenopa binotata* complex. Terms including family, replicate, or individual were random effects. The main family term tests for differences in the average elevation of the preference functions (i.e., in overall responsiveness; Rodríguez et al. 2013b). The key term is the family × stimulus frequency quadratic interaction, which tests for overall variation in the curvilinear aspect of the preference functions (Rodríguez et al. 2013b). Other terms included in the model for completeness. Significant terms indicated in boldface

Term	MS	*F*-ratio (df num, df den)	*P*
*E. binotata “*Ptelea”			
Family	82.1	**2.63** (14, 15.53)	**0.0347**
Replicate	44.3	1.40 (14, 115)	0.1625
Individual	31.6	**10.94** (115, 2562)	**<0.0001**
Stimulus frequency	1469	**508.96** (1, 2562)	**<0.0001**
Stimulus frequency^2^	574.3	**65.92** (1, 15.65)	**<0.0001**
Family × stimulus frequency	23	**7.95** (14, 2575.7)	**<0.0001**
Family × stimulus frequency^2^	9.9	**3.41** (14, 2562)	**<0.0001**
Residual	2.9		
*E. binotata* “Viburnum”			
Family	14	1.6 (18, 21.1)	0.1367
Replicate	11.9	1.3 (19, 180)	0.2021
Individual	9.3	**10.3** (180, 3886)	**<0.0001**
Stimulus frequency	5	**5.5** (1, 3886)	**0.0185**
Stimulus frequency^2^	790	**357.1** (1, 21.0)	**<0.0001**
Family × stimulus frequency	4	**4.4** (18, 3903.5)	**<0.0001**
Family × stimulus frequency^2^	2.5	**2.8** (18, 3886)	**<0.0001**
Residual			

### Testing for genetic variation in female preferences and male signals

**Figure 3:**
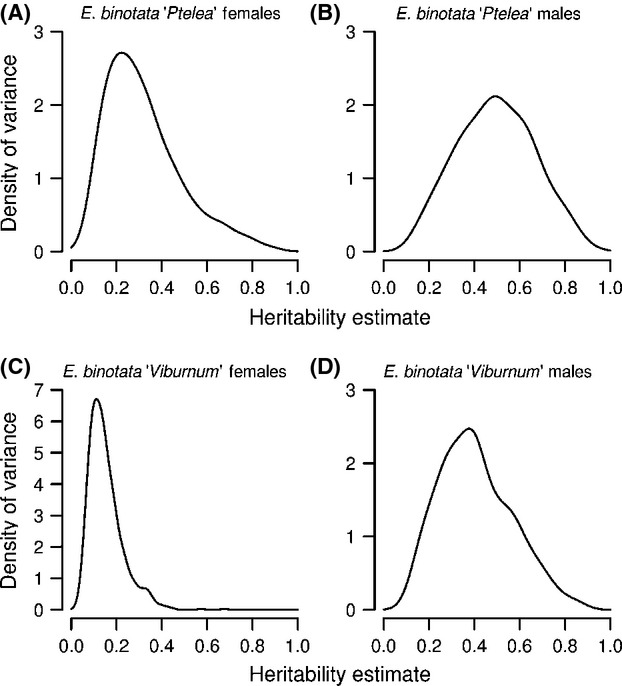
Posterior distributions of heritability estimates for male signals and female preference of *Enchenopa binotata “*Viburnum” (A and B) and *E. binotata “*Ptelea” (C and D). Each distribution is a density curve of heritability estimates from 1000 iterations of an animal model run with 1,000,000 iterations with a burn-in of 500,000 iterations, and sampled every 500. All panels plotted on the same scale except (C).

We found genetic variation in female peak preference (low magnitude) and in male signal frequency (intermediate magnitude) in both species (Table2; Fig.3).

**Table 2:** Animal model estimates and confidence intervals for genetic parameters for female peak preference and male signal frequency in two members of the *Enchenopa binotata* complex. CV_A_ estimates derived from the variances estimated by the animal model

	Peak preference		Signals		Signal–preference genetic correlation (CI)
	Heritability (CI)	CV_A_	Heritability (CI)	CV_A_	
*E. binotata “*Ptelea”	0.20 (0.07–0.67)	2.66	0.50 (0.18–0.82)	2.37	−0.14 (−0.81–0.66)
*E. binotata “*Viburnum”	0.09 (0.04–0.31)	2.84	0.40 (0.12–0.71)	2.23	0.35 (−0.72–0.70)

### Testing for signal–preference genetic correlations

**Figure 4:**
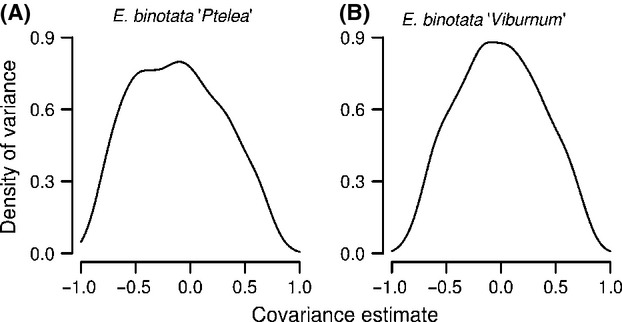
The density of genetic correlation estimates sampled 1000 times across all iterations in the animal model. Posterior distributions of estimates of genetic correlations between male signals and female preference of *Enchenopa binotata “*Viburnum” (A) and *E. binotata “*Ptelea” (B). Each distribution is a density curve of heritability estimates from 1000 iterations of an animal model run with 1,000,000 iterations with a burn-in of 500,000 iterations, and sampled every 500.

The animal model estimated signal–preference genetic correlations of different magnitude and sign for the two species, but in both cases, the CIs overlapped zero (Table2; Fig.4).

**Figure 5:**
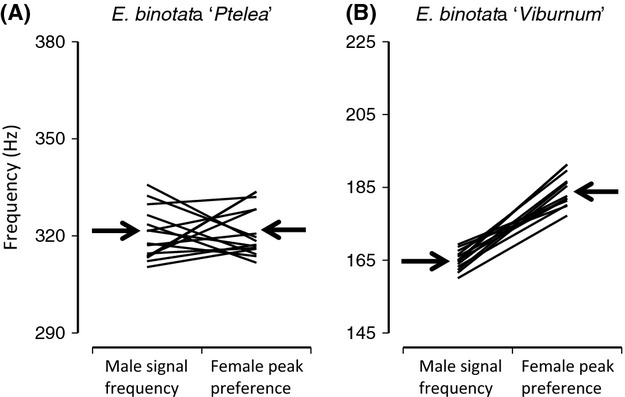
Variation among full-sib families in male signal frequency and the peak of female preferences for signal frequency, in two members of the *Enchenopa binotata* complex. Each line shows one family’s mean values for signal frequency and female peak preference. The inclination of the lines indicates the degree of family-level signal–preference correspondence (perfect correspondence = horizontal line). Arrows indicate overall means pooling across families. The range of the *y*-axes indicates the overall range of phenotypic variation. Results differed between our two study species. (A) For *E. binotata “*Ptelea,” the signal–preference genetic correlation was negative (note line crossovers) but with CIs overlapping zero (Table2). However, some families (those with near parallel horizontal lines) remained distinct from some others in their signal–preference relationship (text; Table3). Note the population-level signal–preference correspondence (arrows). (B) For *Enchenopa binotata* “Viburnum,” lower overall genetic variation and fewer crossovers resulted in a positive signal–preference genetic correlation but with CIs overlapping zero (Table2). Note the population-level signal–preference mismatch (arrows).

The reaction norm approach helped explore differences in the signal–preference relationship that underlie the genetic correlation estimates. For *E. binotata* “Ptelea,” the main family term was nonsignificant and the family × sex interaction was significant (Table3), indicating a signal–preference genetic correlation of *r *<* *1 due to family-level signal–preference mismatch (Fig.5A). Heritability in peak preference was lower than that in signals (Table2), but the range of variation across families was similar for both sexes (Fig.5A). This indicates that the lower preference heritability was due to a greater amount of within-family variation in preference, rather than to a lower among-family spread (i.e., the pattern is closer to Fig.1F than to Fig.1G). The significant family × sex interaction is consistent with the animal model estimate of a weakly negative *r*. However, the family term tested over the residual (*F *= MS_family_/MS_residual_) (Table3) was significant, indicating that some families do not overlap with others in their signal–preference relationship (Fig.5A). The nonsignificant sex term (Table3) indicates population-level correspondence between mean values for signals and preferences (arrows in Fig.5A).

**Table 3:** Exploration of genetic variation in the signal–preference relationship with the reaction norm approach (see text), in two members of the *Enchenopa binotata* complex. In the basic linear mixed model in JMP, the family × sex interaction tests for signal–preference mismatch among full-sib families (signal–preference genetic correlation of *r *<* *1). The family term tests for *r *>* *0 above signal–preference mismatch. By contrast, the “Scheffé model” test of *F *= MS_family_/MS_residual_ asks about differentiation in the signal–preference relationship for some genotypes. All terms involving family or replicate are random. Significant terms in boldface

Species	Term	MS	Linear mixed model		Scheffé model	
			*F*-ratio (df num, df den)	*P*	*F*-ratio (df num, df den)	*P*
*E. binotata “*Ptelea”	Family	527.4	0.66 (14, 19.1)	0.78	**3.11** (14, 269)	**0.0002**
	Replicate	407.1	**2.40** (15, 269)	**0.0028**		
	Sex	24.3	0.05 (1, 14.7)	0.84		
	Family × sex	571	**3.37** (14, 269)	**<0.0001**		
	Residual	169.5				
*E. binotata “*Viburnum”	Family	189.0	1.06 (12, 8.79)	0.47	1.62 (12, 288)	0.085
	Replicate	136.5	1.17 (13, 288)	0.30		
	Sex	25333.4	**164.15** (1, 13.56)	**<0.0001**		
	Family × sex	157.5	1.35 (12, 288)	0.19		
	Residual	116.8				

For *E. binotata* “Viburnum,” the main family term and the family × sex interaction term were both nonsignificant (Table3), indicating low genetic variation in signals and preferences and in the signal–preference relationship (Table2; Fig.5B). Heritability in peak preference was lower than that in signals (Table2), but the range of variation across families was similarly narrow for both sexes (Fig.5B). This indicates that the lower preference heritability was due to a greater amount of within-family variation in preference rather than to a lower among-family spread. The nonsignificant family × sex interaction is consistent with the animal model estimate of a positive *r*. However, the marginally significant test of *F *= MS_family_/MS_residual_ only provides weak evidence that some families do not overlap in the signal–preference relationship (Table3; Fig.5B). The significant sex term (Table3) indicates a population-level mismatch between mean values for signals and preferences (arrows in Fig.5B).

In short, the animal model approach and the “SAS model” in the reaction norm approach give consistent results for signal–preference genetic correlations (Tables3): for *E. binotata* “Ptelea,” a weakly negative *r* estimate (with CIs overlapping zero) and a significant family × sex interaction; for *E. binotata* “Viburnum,” a positive *r* estimate (with CIs overlapping zero) and nonsignificant family × sex interaction. The “Scheffé model” test of *F *= MS_family_/MS_residual_ adds detail to the picture, finding that some families do not overlap in the signal–preference relationship, with stronger evidence of this in the species that showed greater amounts of genetic variation in signals and preferences (*E. binotata* “Ptelea”; Table3; Fig.5).

### Range of signal–preference genetic variation relative to species differences

**Figure 6:**
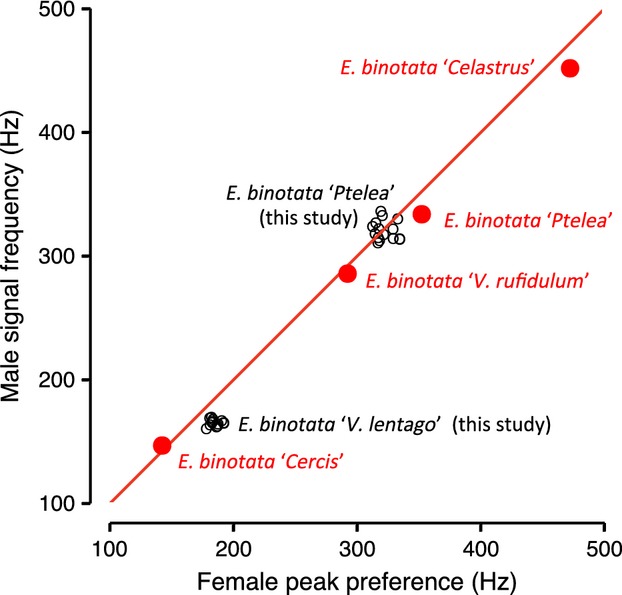
Range of variation in signals and preferences among full-sib families in our two study species, contrasted with the magnitude of species differences in signals and preferences across the *Enchenopa binotata* complex. Red symbols and line: mean values for signal frequency and peak preferences for four sympatric species at the collecting site of one of our study species (from Rodríguez et al. 2006 with permission). Although over 11 species are known to exist in the *E. binotata* complex, these four span the known range of variation in signal frequency in the complex. The line indicates a one-to-one signal–preference relationship. Black symbols: family means for signals and peak preferences for our two study species. Note that in this figure, we distinguish two treehopper species that live on different *Viburnum* hosts in our two study sites (*E. binotata* “Viburnum rufidulum,” which is sympatric with *E. binotata* “Ptelea” in Missouri; and *E. binotata* “Viburnum lentago” in Wisconsin).

The range of within-population (among-family) variation in mean signal and preference values covered a substantial portion of the difference between some species in the *E. binotata* complex (Fig.6). For *E. binotata “*Ptelea,” the most distinct full-sib families in our sample spanned just under 50% of the difference between the species mean and the mean for the most similar sympatric member of the complex (Fig.6). For *E. binotata “*Viburnum,” the most distinct families in our sample spanned just under 30% of this species difference (Fig.6).

## Discussion

We examined signal–preference genetic correlations in two members of the *E. binotata* complex of treehoppers to ask about the potential of Fisherian selection to explain signal–preference coevolution. In both study species, the signal–preference genetic correlation was (at best) weak. Nevertheless, there was also indication that some genotypes were sufficiently distinct from some others in their signal–preference relationships that signal–preference coevolution may be promoted. Consider that, although many genotypes in Figure5A cross with each other, some genotypes do not, and may thus mate assortatively. The phenotypic difference between these extreme genotypes may be evolutionarily important, as it spanned up to nearly half the distance between some extant species in the *E. binotata* complex (Fig.6).

The above indication of signal–preference differentiation among a subset of genotypes was stronger in the species that showed higher heritabilities in signals and preferences (*E. binotata* “Ptelea”). This is in agreement with the basic requirement of genetic variation in signals and preferences for signal–preference genetic correlations to be established (Fisher 1930; Bakker and Pomiankowski 1995; Roff and Fairbairn 2014). Note also that this was in spite of the fact that this species also showed greater signal–preference mismatch among genotypes. Thus, we suggest that there may be some forms of the signal–preference relationship that are more likely than others to promote coevolution by Fisherian selection, even if the genetic correlation is overall weak or absent (cf. Prum 2010).

Species differences in the amount of genetic variation expressed in signals and preferences may have several explanations. One potential factor is hinted at by the observation of population-level signal–preference correspondence for *E. binotata* “Ptelea” but of mismatch for *E. binotata* “Viburnum.” This result suggests stabilizing sexual selection on signal frequency arising from mate choice for *E. binotata* “Ptelea,” but directional selection for *E. binotata* “Viburnum” (cf. Rodríguez et al. 2006). We have no indication that the strength of these putative stabilizing and directional forms of selection would vary. However, we speculate that directional selection in *E. binotata* “Viburnum” might be ongoing, while the observed stabilizing selection in *E. binotata* “Ptelea” might represent an older divergence event, so that genetic variation in *E. binotata* “Ptelea” may have had more time to become replenished. An alternative explanation for species differences in the expression of genetic variation might involve differences in the experimental conditions used to estimate the components of variation (Roff 1997; Lynch and Walsh 1998; Sgrò and Hoffmann 2004). However, this possibility is unlikely in this study, due to our standard procedures. These questions remain of interest for understanding variation in the presence and magnitude of signal–preference genetic correlations (Bakker and Pomiankowski 1995; Fowler-Finn and Rodríguez 2015).

Fisherian selection is a simple mechanism that may explain signal–preference coevolution among diverging populations and recently diverged species (Fisher 1930; Lande 1981; Kirkpatrick 1982; Higashi et al. 1999; Mead and Arnold 2004). The biological relevance of Fisherian selection depends in part on how common signal–preference genetic correlations are in nature. Recent work indicates that, once key predictors of the likelihood of the establishment of signal–preference genetic correlations are accounted for, these correlations seem to be more common than previously anticipated (Fowler-Finn and Rodríguez 2015). Additionally, here we argue that even some forms of signal–preference relationship when correlations are weak or absent may promote coevolution, due to assortative mating among subsets of genotypes. Assessing the relevance of such patterns will require further investigation with robust quantitative implementations of our qualitative tests using broad comparative samples. Important additional questions will involve how the evolutionary processes that arise from direct signal–preference genetic correlations may interact with additional factors that reinforce the action of Fisherian selection (e.g., Bailey and Moore 2012; Chandler et al. 2012; Rebar and Rodríguez 2013, 2014a,b, 2015; Greenfield et al. 2014), and with factors that oppose it (Kirkpatrick and Ryan 1991; Servedio and Bürger 2014). Nevertheless, there seems to be good reason to expect that Fisherian selection may often make important contributions at the beginning of divergence (Fisher 1930; Prum 2010).
